# Benefits of interventions for respiratory secretion management in adult palliative care patients—a systematic review

**DOI:** 10.1186/s12904-016-0147-y

**Published:** 2016-08-09

**Authors:** Juliano Ferreira Arcuri, Ebun Abarshi, Nancy J. Preston, Jenny Brine, Valéria Amorim Pires Di Lorenzo

**Affiliations:** 1 Federal University of São Carlos, Rod Washington Luiz, km 235, Monjolinho, São Carlos, SP CEP 13565-905 Brazil; 2 International Observatory on End of Life Care, Faculty of Health and Medicine, Furness College, Lancaster University, Bailrigg, LA1 4YG UK; 3 Lancaster University Library, Lancaster University, Bailrigg, LA1 4YG UK

**Keywords:** Respiratory secretion, Palliative Care, Cough, Sputum

## Abstract

**Background:**

Respiratory secretions impact negatively on palliative patients. Unfortunately, a gold standard therapy is not yet available. The purpose of this study was to identify which interventions are in use to control respiratory secretions in patients with chronic disease with a poor prognosis and verify their effects on outcomes relevant for palliative care patients.

**Methods:**

A systematic review of the literature with narrative summary was conducted. We searched eight electronic databases in April ^6th^, 2016. Citation-tracking and reference list searches were conducted. We included randomized controlled trials, crossover trials, observational and qualitative studies regarding interventions for respiratory secretion management in adult patients with chronic diseases that met inclusion criteria indicating short prognosis.

**Results:**

Six randomized controlled trials, 11 observational studies, ten crossover trials and one qualitative study were found. Interventions included mechanical insufflation-exsufflation (MIE), expiratory muscle training, manually-assisted cough, tracheotomy, chest physiotherapy, suctioning, air stacking, electrical stimulation of abdominal muscles, nebulized saline, positive expiratory pressure masks, percussive ventilation, high frequency chest wall oscillations. The interventions with most promising benefits to patients in palliative care were manually-assisted cough and mechanical insufflation-exsufflation to promote expectoration and percussive ventilation to improve mucous clearance.

**Conclusion:**

Therapies, such as manually assisted cough, mechanical insufflation-exsufflation and percussive ventilation, which aim to deal with respiratory secretion, were the most promising treatment for use in palliative care for specific diseases. Nevertheless, the evidence still needs to improve in order to identify which treatment is the best.

**Electronic supplementary material:**

The online version of this article (doi:10.1186/s12904-016-0147-y) contains supplementary material, which is available to authorized users.

## Background

The presence of mucus within and around the respiratory tract towards the end-of-life can be burdensome, for patients already facing death for a number of reasons. Continuous accumulation of mucus tends to negatively impact the quality of life and the dying process and leads to social isolation as excessive expectoration may be disturbing to some people and cultures [1]. However research around the benefits of interventions used to manage this symptom is somewhat scarce. Problems in dealing with respiratory secretions are caused by increased production of airway secretions or inefficient elimination of the mucus or both and as a result, mucus encumbrance is difficult to control.

The increased production of respiratory mucus is common in patients with cardiorespiratory [2] diseases and lung, head and neck cancer. Sometimes these conditions are associated with cough inefficiency due to muscle weakness and poor coordination [3, 4]. However, other diseases may cause this discomfort only due to a deficiency in mucus elimination caused by cough inefficiency such as in neurologic diseases [1, 5–7]. Most of the diseases associated with the presence of respiratory secretion are chronic conditions and they may be life-threatening in their later stages, which is an indication for palliative care.

As a consequence to patients, an augmented amount of secretion is associated with an increase in dyspnoea, cough and the chances of respiratory complications, such as pneumonia. Furthermore, there is an associated poor prognosis in chronic obstructive pulmonary disease (COPD) patients [3, 4] and secretions may interfere in the efficacy of other interventions, such as non-invasive ventilation. Unfortunately, a gold standard therapy is not yet available.

The interventions to help patients deal with respiratory secretion have different goals and the choice must depend on the patient’s condition. Three main goals are: (1) promote expectoration; (2) increase mucociliary clearance and conduct of the secretions to the upper airways; (3) improve cough effectiveness.

Some therapies that are suitable to treat patients with reversible conditions that are facing difficulties in dealing with respiratory secretion, might not be suitable in patients in palliative care. Suctioning is one such therapy indicated to remove respiratory secretion from patient’s airways, however, it is uncomfortable and associated with complications, such as pain, uncontrollable coughing, infection, atelectasis, hypoxemia, haemoptysis and airway injuries [8]. In addition, palliative care guidelines suggest that the indication of suctioning procedure should be done with caution, since it is a painful procedure [9, 10].

Other pharmacological and non-pharmacological interventions have been clinically used to control respiratory secretion and examples are mucolitics [11], antibiotics [3] and respiratory physiotherapy [1, 12]. Systematic reviews [11, 13] have shown that mucolitics and respiratory physiotherapy have been successfully used in the management of this symptom in patients with COPD, but not specifically in the end stages.

However, there is no clear evidence on whether the available interventions would be effective and suitable for patients receiving palliative care, since patients facing life-threatening diseases have specific treatment goals, which are not always verified in studies with patients with less severity.

### Research question

What are the benefits of pharmacological and/or non-pharmacological interventions in adult palliative care patients that are facing problems in dealing with respiratory secretion?

### Objective

The objective of this study was to identify which interventions are in use to control respiratory secretion in patients with chronic disease with a poor prognosis and verify their effects on outcomes relevant for palliative care.

## Methods

### Search strategy and data extraction

The literature search was conducted in September 23th of 2014, and updated in April 6^th^ of 2016. The review process involved:

1) A literature search was performed to retrieve journal articles and grey literature using the search strategy (Additional file 1). Thereafter, the results were imported to the reference software ENDNOTE X7® (Thomson Reuters, New York, NY) (JB) 2).2) An initial selection based on titles was performed by two independent reviewers (JFA and JB) and possible disagreements were resolved by a third reviewer (VAPDL). Studies were excluded when they were duplicates or clearly did not meet the inclusion criteria (Table 1) 3) The abstracts of these studies were read and retained where the content was relevant to the topic under consideration (JFA and JB) with disagreements resolved by a third reviewer (VAPDL) 4) The full papers were read by two independent reviewers (JFA and EA) to verify the inclusion criteria 5) A citation track and a reference search of included studies was performed (JB) 6) Data extraction was performed by two independent reviewers (JFA and EA) 7) The assessment of the methodological quality of the studies was done by two independent reviewers (JFA and VAPDL) using the PEDro scale, a 11-item scale, which ten are included in the total score, in which higher scores represent better methodological quality [14, 15]. Lastly, the evidence was summarized using the Oxford Centre for Evidence-based Medicine (OCEBM) levels of evidence 2011, this score considered the level of evidence as (1) systematic reviews of randomized controlled trials; (2) Randomized controlled trials or observational studies with dramatic effect; (3) Non-randomized trials/follow-up studies; (4) case series, case-control studies or historically controlled studies and (5) Mechanism-based reasoning [16]. The level is recorded in brackets against the studies.

**Table 1:** Inclusion criteria

Inclusion	Exclusion
Was the population studied with the severity of the chronic disease identifiable and with one of the National Hospice Organization Criteria? [15]	Did the study involve a population with disease severity that is not identifiable, or without any of the National Hospice Organization Criteria? [15]
Were the interventions performed to treat cough ineffectiveness, respiratory hypersecretion or its consequences (relative distress)?	Were the interventions focused in treating other symptoms?
Was the study composed in its majority of adults?	Did the study involve animals?
Was it a randomized clinical trial, observational study or a qualitative study?	Was the study about death rattle?

Ethics approval was not requested since this study did not involve humans, human data or animals.

### Electronic search

The following databases were searched: AMED, British Nursing Index, CINAHL, EMBASE, LILACS (for South American publications), PEDro, MedLine, Web of Science. The search carried out in MedLine is set out in the table in the Additional file 1 and was adapted for use in other databases.

### Grey literature

Searches were carried out in clinical trials registers, including the Cochrane Airways Group Specialised Register of trials, thesis and dissertations databases, NICE Evidence, GoogleScholar (JB). The studies retrieved were evaluated by two different reviewers (JFA and NP) and any disagreement was resolved by a third reviewer (VAPDL).

### Outcomes

The primary outcomes of the review were subjective impression on effectiveness of the intervention and comfort during therapy. Moreover, all outcomes used to assess the therapies in the studies were included. The outcomes were presented as mean ± standard deviation or median (interquartile range 25–75 %). Based in their opinion, authors classified the outcomes as critically relevant, relevant or less relevant for clinical decision in palliative care.

## Results

**Fig. 1:**
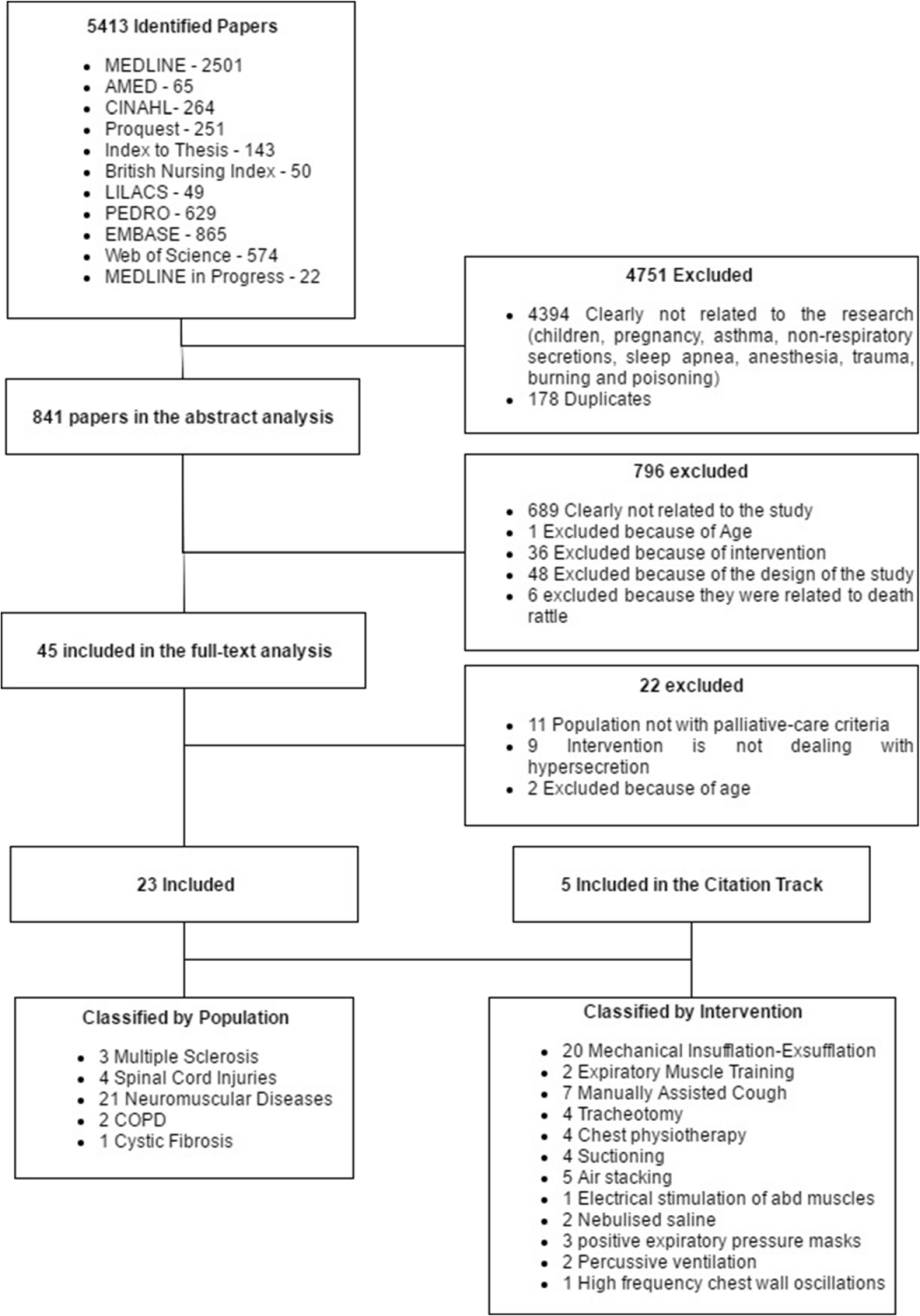
PRISMA Flowchart

The search was conducted in September 2014 and updated in April 2016. 5413 papers were identified by the search which resulted in 28 studies included in the review, a study flowchart is on Fig. 1. These included six randomized controlled trials [17–22] (Table 2), ten cross-over trials [23–32] (Table 3), 11 observational studies [33–43] and one qualitative study [44]. Associated conditions were multiple sclerosis [18, 21, 36], neuromuscular disorders [19, 22–29, 31, 33–36, 38–44], spinal cord injury [17, 30, 35, 37], COPD [20, 24] and cystic fibrosis [32]. Interventions were mechanical insufflation-exsufflation (MIE) [17, 22–29, 33–44], manually assisted cough (MAC) [23, 25, 28, 29, 31], expiratory muscle training (EMT) [18, 21], percussive ventilation [31], positive expiratory pressure (PEP) masks associated [32] or not [20] to hypertonic saline nebulisation, abdominal muscles electrical stimulation [30] and vibratory vest [19] (Table 4). Other interventions were mentioned in the studies which were represented as usual care but, since they were used alongside other interventions, they lacked results for evidence summarization.

**Table 2:** Randomized clinical trials

Population\PEDro Scale	Groups	Intervention	Outcomes
Pillastrini et al (2006) [17] Upper Spinal Cord Injury 1	Control (*n* = 4)	Chest Physiotherapy	No improvement: FVC; FEV_1_; PCF; FEV_1_/FVC; PaO_2_; PaCO_2_; Ph; SaO_2_.
	Experimental (*n* = 5)	Chest Physiotherapy + MIE	Improvement: FVC^a^ (Before 0.37 ± 0.23 ml /After 0.46 ± 0.21;↑24 %); FEV_1_^a^ (Before 0.21 ± 0.15 ml /After 0.28 ± 0.14; ↑33 %); PCF^a^ (Before 0.24 ± 0.19 L/s /After 0.31 ± 0.19 L/s;↑29 %). No improvement: FEV_1_/FVC; PaO_2_; PaCO_2_; Ph; SaO_2_.
Gosselink et al (2000) [18] Multiple Sclerosis 5	Control (*n* = 9)	Non-supervised breathing exercises	No Improvement: PIMax; Pulmonary Index, FVC; PEMax.
	Training (*n* = 9)	Expiratory muscle training (Threshold)	Improvement : PIMax^a^ (↑39 ± 41cmH_2_O); Pulmonary Index^b^ (↓ 2 ± 1pts). No Improvement: FVC; PEMax.
Chaisson et al (2006) [19] ALS 6	Control (*n* = 4)	Manually assisted cough	No improvement: Respiratory complications; Rate of decline in FVC; Survival days.
	Experimental (*n* = 5)	Manually assisted cough + Vibratory vest	No improvement: Respiratory complications; Rate of decline in FVC; Survival days.
Christensen et al (1990) [20] COPD 4	Control (*n* = 22)	SHAM therapy with low PEP mask	No improvement: FEV_1_; FVC; PaO_2_; PaCO_2_; dyspnoea during activities; cough; sputum; exacerbations; bedridden days; hospitalizations and days with antibiotics.
	Experimental (*n* = 25)	Therapy with PEP mask	No Improvement: FEV_1_; FVC; PaO_2_; dyspnoea walking on ground level; sputum; exacerbations; bedridden days; hospitalizations and days with antibiotics. Worsening: Cough^b^ [↓11 (−69–75)mm]; PaCO_2_ [↑0.05(−69–75)kPa]^b^; dyspnoea walking on staircase^b^ [↓1 (−69–53)mm].
Smeltzer et al (1996) [21] Multiple Sclerosis 4	Control (*n* = 5)	SHAM therapy with Threshold	No improvement: PIMax; PEMax.
	Experimental (*n* = 10)	Expiratory muscle training (Threshold)	Improvement: PEMax^b^ (19,4 ± 9.9cmH_2_O; ↑19 %). No improvement: PIMax.
Rafiq et all (2015) [22] NMD 5	Breath Stacking (*n* = 21)	Breath Stacking Therapy	No improvement: Hospital Admissions, number of antibiotics days, pulmonary morbidities, Survival, quality of life.
	MIE (*n* = 19)	MIE	No Improvement: Hospital Admissions, number of antibiotics days, Hospital Admissions, number of antibiotics days, pulmonary morbidities, Survival, quality of life.

Values presented as mean ± standard deviation or median (interquartile range). ^a^pre-post intra-group comparison; ^b^statistical difference between groups; *FVC* forced vital capacity, *FEV*_*1*_ forced expiratory volume in the first second, *PCF* peak cough flow, *PaO*_*2*_ Arterial pressure of oxygen, *PaCO*_*2*_ Arterial pressure of carbon dioxide, *SaO*_*2*_ Arterial Oxygen Saturation, *MIE* Mechanical Insufflation-Exsufflation, *PIMax* Maximum inspiratory pressure, *PEMax* Maximum expiratory pressure, *COPD* Chronic obstructive pulmonary disease, *PEP* Positive expiratory pressure, *ALS* Amyotrophic lateral sclerosis, *NMD* Neuromuscular Disease

**Table 3:** Cross-over trials

Population/PEDro Scale	Interventions	Outcomes		
Senent et al (2011) [23] Neuromuscular diseases (*n* = 16) 3	Coached unassisted cough	No improvements		
	Couched unassisted cough + abdominal thrust	No improvements		
	Abdominal thrust + Air Stacking	Improvements: PCF:284(146–353)L/min, ↑238 % than baseline 84(35–118)L/min (statistically higher than first two methods)		
	Abdominal thrust + usual patient’s bi-level ventilator	Improvements: PCF:212(99–595)L/min, ↑152 % than baseline (statistically higher than first two methods)/Comfort (VAS): 8(7–8)pts,↑60 % than baseline 5(4–7)pts (statistically higher than all other methods).		
	Abdominal thrust + IPAP of +30cmH_2_O	Improvements: PCF: 233(100–389)L/min, ↑177 % than baseline (statistically higher than first two methods).		
	MIE (40cmH_2_O)	Improvements: PCF: 488(243–605)L/min (↑480 %) than baseline/Effectiveness (VAS): 8(6–8)pts ↑100 % than baseline 4(2–7)pts (statistically higher than all other methods).		
		NMD (*n* = 7)	ALS (*n* = 13)	COPD (*n* = 9)
Winck et al (2004) [24] Neuromuscular Disease and COPD (*n* = 29) 3	MIE (15cmH_2_O)	No Improvements	No Improvements	No Improvements
	MIE (30cmH_2_O)	No Improvements	No Improvements	No Improvements
	MIE (40cmH_2_O)	Improvements: PCF: 220(190–300)L/min, ↑22 % than baseline 180(150–275)L/min/SpO_2_: 94(92–96), than 98(97–98), ↑4 %/Dyspnoea (BORG) 0.75(0–2.3)pts, ↓62 % than baseline 2(0.4–3.3)pts.	Improvements:PCF:200(170–352)L/min ↑17 % than baseline 170(128–300)L/min/SpO_2_: 98(97–98)%,↑4 % than baseline 94(94–95)%	Improvements: SpO_2_: 97(95–97)%, ↑5 %/ than baseline 92(91–94)%, Dyspnoea (BORG) 1(1–2.5)pts, ↓200 % than baseline 3(2–4)pts.
Bach (1993) [25] Neuromuscular diseases (*n* = 21) 4	Air Stacking	Improvements: PCF: 3.37 ± 1.07 L/s, ↑86 % than baseline 1.81 ± 1.03 L/s		
	Manually Assisted Cough	Improvements: PCF: 4.27 ± 1.29 L/s, ↑135 % than baseline		
	MIE (Individualized pressure)	Improvements PCF: 7.47 ± 1.02 L/s, ↑312 % than baseline (higher than all other methods)/FVC: 0.54 ± 0.39 L, ↑10 % than baseline 0.49 ± 0.37 L/FEF25-75 %: 0.91 ± 0.69 L/s, ↑13 % than baseline 0.80 ± 0.59 L/s		
Sancho et al (2003) [26] ALS (*n* = 6) 5	Tracheal Suctioning	Improvements: WB: 0.95 ± 0.23 J/L, ↓7 % than baseline 1.03 ± 0.25 J/L Worsening: All patients referred as less comfortable and effective than MIE		
	MIE (40cmH_2_O)	Improvements: SpO_2_:,↑3 % than baseline 93.5 ± 2.25 %/Peak Inspiratory Pressure: 15.33 ± 4.13cmH_2_O, ↓17 % than baseline 18.5 ± 4.23cmH_2_O/Mean Airway Pressure: 3.83 ± 1.72cmH_2_O, ↓8 % than baseline 4.67 ± 1.37cmH_2_O/WB: 0.87 ± 0.26, ↓15 % than baseline		
Population/PEDro Scale	Interventions	Outcomes		
Chatwin, et al (2009) [27] Neuromuscular diseases (*n* = 8) 4	Chest Physiotherapy + MIE	Improvements: ↓ Duration of the intervention (17 min shorter than only Physiotherapy)		
Chatwin et al (2003) [28] Neuromuscular Diseases (*n* = 14) 3	Standard Physiotherapy Assisted Cough	No improvements		
	Cough + Non-Invasive Ventilation	No improvements		
	Exsufflation Assisted Cough	Improvements: PCF ↑^a^		
	MIE	Improvements: PCF ↑^a^		
Lacombe, et al (2014) [29] Neuromuscular Disease (*n* = 18) 5	MIE (highest tolerable pressure)	Improvements: PCF ↑ ^a^ than baseline; Effective Cough Time ↑^a^ compared to baseline		
	MAC + IPAP	Improvements: Effectiveness: 8.3(7.2–9)pts, ↑29.6 % than MIE 6.4(4.8–8.2)pts; PCF ↑^a^; Effective Cough Time ↑ ^a^ (all compared to MIE)		
	MAC + MIE (highest tolerable pressure)	Improvements: Effectiveness: 8.5(6.2–9)pts, ↑32.8 % higher than MIE; PCF ↑^a^; Effective Cough Time↑^a^ (all compared to MIE)		
Linder (1993) [30] Patients with quadriplegia (*n* = 8) 5	Cough with FES	Improvements: PEMax: 60 ± 22.8cmH_2_O, ↑119,7 % than baseline 27.3 ± 6.4cmH_2_O		
	MAC	Improvements: PEMax: 83 ± 18.7cmH_2_O, ↑ 38.3 % than Cough with FES		
Toussaint et al (2003) [31] Duchenne dystrophy patients (*n* = 8) 4	Cough assistance techniques	No improvements		
	Percussive Ventilation + cough assistance techniques	Improvements: Removed secretion: 6.53. ± 4.77 g,↑42.8 % than Cough assisted techniques 4.57 ± 3.5 g; (Only in a sub-group of five hypersecretive patients)		
O’Connell, et al (2011) [32] Cystic Fibrosis (*n* = 4) 4	Hypertonic Saline using Jet Nebulizer	No improvements		
	Hypertonic Saline using PEP mask	Improvements: Subjective report of chest tightness 1.7pts, ↓68 % than without PEP mask 5.3pts		

Values presented as mean ± standard deviation or median (interquartile range). Insufflation-Exsufflation; *MAC* Manually assisted cough, *NMD* Neuromuscular Disease, *ALS* Amyotrophic lateral sclerosis, *COPD* Chronic obstructive pulmonary disease, *SpO*_*2*_ Peripheral oxygen saturation, *FVC* Forced vital capacity, *FEF25–75 %* Mean forced expiratory flow at 25–75 % expiratory period, *WB* Work of breath, *FES* Functional electrical stimulation, *PEMax* Maximum expiratory pressure, *PEP* Positive expiratory pressure. ^a^Results presented graphically in the original paper

**Table 4:** Evidence summary

Intervention	Disease	Critically relevant for clinical decision											Relevant for Clinical Decision						Less Relevant for Clinical Decision				
		Comfort	Effectiveness	Health related quality of life	Survival	Resting dyspnoea	Hospital days	Hospital admission	Intervention duration	Respiratory complications	Tracheotomy prev/removal	Cough	Oxygenation	Dyspnoea during exercise	PCF	FEV1	FVC	Secretion Amount	PaCO2, Ph	Chest Tightness	Airway Resistance	PIMax	PEMax
Mechanical insufflator-exsufflator	COPD	---	---	---	---	↓ 3	---	---	---	---	---	---	↑ 3	---	↔ 3	---	---	---	---	---	---	---	---
	SCI	---	---	---	---	---	---	↔ 3	---	↔ 3	---	---	↔ 3	---	↑ 3	↑ 3	↑ 3	---	↔ 3	---	---	---	---
	NMD	↑ 1^a^	↑ 3	↔ 2	↑2	↓ 3	↔ 2	↕ 2	↓ 3	↕2	↑2	---	↑ 3	---	↑ 1^a^	---	↑3	---	---	---	---	---	---
EMT	MS	---	↑ 2	---	---	---	---	---	---	---	---	---	---	---	---	---	---	---	---	---	---	↕ 2	↕ 2
MAC	NMD	↑3	↑ 2	---	---	---	---	---	---	---	---	---	---	---	↑ 2	---	---	---	---	---	---	---	↑3
PEP Mask	COPD	---	---	---	---	---	↔ 2	---	---	↔ 2	---	↑ 2	↔ 2	↕ 2	---	↔ 2	↔ 2	↔ 2	↑ 2	---	---	---	---
	CF	---	---	---	---	---	---	---	---	---	---	---	---	---	---	---	---	---	---	↓3	---	---	---
FES	SCI	---	---	---	---	---	---	---	---	---	---	---	---	---	---	---	---	---	---	---	---	---	↑3
Percussive ventilation	NMD	---	---	---	---	---	---	---	---	---	---	---	---	---	---	---	---	↑ 2	---	---	↔ 2	---	---
Vibratory vest	NMD	---	---	---	↔ 3	---	---	---	---	↔ 3	---	---	---	---	---	---	↔ 3	---	---	---	---	---	---
Tracheotomy	NMD	---	---	---	↑2	---	---	---	---	---	---	---	---	---	---	---	---	---	---	---	---	---	---

OCEBM levels of evidence: 1- Systematic reviews / 2- Randomized controlled trials or observational studies with dramatic effect / 3: Non-randomized trials/follow-up studies

*PCF* Peak cough flow, *FEV*_*1*_ Forced expiratory volume in the first second, *FVC* Forced vital capacity, *PaCO*_*2*_ Arterial pressure of carbon dioxide, *PIMax* Maximum inspiratory pressure, *PEMax* Maximum expiratory pressure, *COPD* Chronic obstructive pulmonary disease, *SCI* Spinal cord injury, *NMD* Neuromuscular disease, *EMT* Expiratory muscle training, *MS* Multiple sclerosis, *MAC* Manually assisted cough, *PEP* Positive expiratory pressure, CF Cystic fibrosis, *FES* Functional electrical stimulation; ↑: increase; ↓: decrease; ↕: conflicting results; ↔: No change. ^a^Considered the systematic review from Morrow and colleagues [46]

The randomized controlled trials and cross-over trials had their methodological quality assessed by PEDro Scale. The highest score (6 points) was achieved by Chaisson et al randomized controlled trial, while the lowest score (1 point) was achieved by Pillastrini et al. [17] Only one study [19] achieved more than a half of the criteria. Five of the six randomized controlled trials did not conceal the random allocation, none were blinded, and one was analysed in intention to treat. Furthermore, only 50 % of the cross-over trials were randomized, none had a concealed allocation, and only one was blinded to the assessors [27]. Although the sample size is not assessed by PEDro scale, three randomized controlled trials do not present power calculation [17, 19, 21]. The scores in PEDro scale are in Tables 2 and 3, and a detailed table is available in the Additional file 1 Table S2 and Table S3).

### Therapies to promote expectoration

Three therapies were found to help promoting expectoration (Table 4): MAC, tracheotomy (to facilitate suctioning procedures) and MIE. MAC and tracheotomy are usual procedures in patients with cough inefficiency. However, MIE equipment is not usual in general hospitals, but is relatively common in neuromuscular disease care centres.

MAC is a technique which increases peak cough flow by manual thrust in the abdominal wall conducted by a therapist or carer that can be optimized using some sort of inspiratory aid, such as a resuscitation bag or mechanical ventilation [23]. It is a simple and relatively cost-free intervention and should be the first line therapy in cough inefficiency. Nevertheless, patients must have a minimum level of cooperation; otherwise they would not cough with the manual thrust [45] and in very severe patients, it might cause fatigue. Moreover, abdominal thrust may cause pain in some cancer patients.

MAC (Tables 2 and 3) was considered more comfortable than other techniques (level 3) [23] and led to an increase in maximum expiratory pressure (PEMax) (level 3) [30] and peak cough flow (PCF) (level 2) [23, 25, 29] and in two studies, the increase was high enough to achieve a level of PCF (>270 L/min) considered to be effective [23, 29]. In addition, effectiveness was confirmed by the patient’s subjective impression (level 3) [23, 29].

However, one study presented contradictory results [28] for PCF, comfort and effectiveness. This study used a non-invasive mechanical ventilator for inspiratory assistance and pressure was adjusted according to patient comfort, while the studies with better outcomes used the highest tolerable pressure or 40cmH_2_O [29] or air stacking using a resuscitation bag [23, 25]. This contradiction suggests that the inspiratory aid should always be the maximum tolerated or 40cmH_2_O. This finding is corroborated by Senent and colleagues [23] who tested both inspiratory aid of 30cmH_2_O and resuscitation bag and found that the former was not as effective as the latter.

Another intervention was tracheotomy, which is a common procedure and associated with mechanical ventilation which can increase survival (level 2) in patients with cough insufficiency (amyotrophic lateral sclerosis and Duchenne dystrophy) [39, 40], since it facilitates and makes suctioning and ventilatory assistance more tolerable. However, a surgical procedure is not usually the choice in patients in palliative care, unless there is no other non-invasive alternative. Moreover, tracheotomy might impair speaking resulting in lower social interaction and swallowing (the tube weight decreases the movements of the trachea, which changes the dynamics of swallowing), which might not be compensated by the gain in survival time.

The therapy with the most included studies was MIE (Tables 2 and 3), where a patient receives a positive pressure, by a mask or tracheotomy, to inflate the lungs, followed by a negative pressure to promote expectoration. Twenty one studies assessed MIE (two randomized controlled trial, one qualitative study, seven cross-over trials and 11 observational studies). A cross-over trial verified the effect of this therapy in COPD and neuromuscular diseases [24]; one verified its effects in a group of patients with respiratory failure with different illnesses [35], all the other studies included patients with neurological [17, 37], neuromuscular [22, 23, 26, 27, 29, 33, 34, 36, 38–44], or both diseases [25, 28].

MIE is a promising therapy to promote expectoration in palliative care. Firstly, it is more comfortable than suctioning (level 3), but this was only studied in tracheotomised patients [26] and comfort might be even better in comparison with nasotracheal suctioning with MIE by a mask which future studies need to confirm. In addition, the use of MIE associated with non-invasive ventilation may prevent, delay or allow the removal tracheotomy in patients with NMD (level 2) [33, 35, 43], decrease respiratory complications [42] and hospitalizations [36]. A study [22] verified no changes in hospitalization, antibiotics days, pulmonary moridities using MIE when compared to cough assisted by breathing stacking technique. However, this study had more severe patients included in MIE group when compared to breath stacking group, which may have influenced the results.

Secondly, according to patients’ subjective assessments, MIE was more effective than suctioning (level 3) [26] and, when associated to MAC, MIE was more effective than MAC alone [23, 29]. In a randomized controlled trial, Pillastrini and colleagues [17] verified an increase in PCF which corroborates the effectiveness findings. Moreover, increase in PCF values was verified by other studies and a systematic review, and it increased above 270 L/min (level 1) [23, 25, 29, 46]. Furthermore, MIE improves oxygenation in COPD and NMD patients [24].

Thirdly, MIE is a therapy that can be conducted by family carers and it brought the feeling that they were able to do something for the patient (level 4) [44]. It may be one of the factors why MIE along with other non-invasive ventilation approaches reduced the number of patients with Duchenne Dystrophy that were living in a rehabilitation hospital [42]. Nevertheless, MIE is contra-indicated when patients present increased risk of pneumothorax, which might happen in patients with some cancers, such as sarcoma, lung carcinoma, germ cell tumor or lymphoma [47].

### Therapies to facilitate mucus clerance

Before providing aid to promote expectoration, a therapist should perform some interventions to help the mucus collection from peripheral areas of the lung, to central airways, such as the trachea, where a cough or suctioning are able to remove the secretions from the airways.

Although chest physiotherapy is the most commonly used therapy, it was only present in studies as a control therapy; there were no studies that compared it to no-treatment in patients in palliative care. Moreover, what was considered as chest physiotherapy was not the same in each study, and may have included manual vibration, postural drainage, manual or mechanical hypersinsuflation, chest compressions, PEP masks and suctioning. Nevertheless, two studies [17, 27] assessed the pre and post effects of chest physiotherapy (as a control therapy). They verified that chest physiotherapy was as effective as chest physiotherapy plus MIE, however the latter required more therapy time [27]. Moreover, chest physiotherapy improved arterial pressure of oxygen in 32 % of participants, but the result was not statistically significant, probably due to the small sample size (*n* = 5) [17]. In addition, some evidence was found regarding other interventions with the same goal, such as PEP [20, 32], vibratory vest [19], percussive ventilation [31]..

The therapy using PEP masks (Tables 2 and 3) improves mucus collection as it maintains the opening of peripheral airways during a forced expiration longer than without the device, which may influence the mucus clearance by increasing the period of expiratory flow. However, only one study was found in very severe COPD patients [20] and another in cystic fibrosis with very severe obstruction [32], which fitted palliative care criteria. In patients with COPD, this therapy may increase the discomfort caused by cough and dyspnoea while walking up a staircase (level 2) [20]. Nevertheless, in patients with cystic fibrosis, PEP masks associated with saline jet nebulizers improved the chest tightness subjective feeling when compared to only saline jet nebulisation (level 3) [32]. However, in the authors’ opinion, since chest tightness is a less relevant outcome (an adverse effect of hypertonic saline nebulisation), the use of PEP mask are not recommended in both diseases until more evidence is found.

The therapy with oscillations or vibrations is based on the effects on increasing the mucociliary function, which mobilizes the secretions from peripheral airways and alter the mucus rheology [19]. Two ways to provide oscillations were found in the review, which were high frequency chest wall oscillations, commonly known as a vibratory vest (Table 2) and percussive ventilation (Table 3). The first method is a vest that inflates until the thorax is gently pressed. When the device is turned on, it inflates and deflates in a determined frequency (5-20Hz) that produces a vibration in the chest wall that is transferred to the airways and mucus. This method was assessed in amyotrophic lateral sclerosis (ALS) patients and failed to improve survival, rate of forced vital capacity (FVC) loss and the number of respiratory complications (level 3) [19]. However, some problems in the study, such as the difference in age between groups and the small sample (*n* = 4 in control group and *n* = 5 in experimental group), are issues that prevent a better judgment in the use of the therapy. The percussive ventilation presented more promising results, resulting in a higher amount of secretion suctioned after the procedure, compared to usual care (level 3) [31]. Percussive ventilation is a therapy in which air pressure is provided by a device by a mask, endotracheal tube or tracheotomy and this pressure oscillates generating a vibration directly in the airways.

### Therapies to improve cough efectiveness

Some therapies are intended to help the individual to regain cough function, when this reaction is lost for some reason. Usually, it requires activating the expiratory muscles by an external stimulus [30] or increasing the expiratory muscle strength [18, 21]. When patients still have muscle mass, but are not able to activate it due to neurological impairment, such as spinal cord injury, functional electrical stimulation (FES) (Table 3) may help improve cough effectiveness by activating the abdominal muscles that were not functional [30]. One cross-over trial, with a small sample size, verified the use of FES in spinal cord injury and verified an increase in PEMax compared to control (level 3) [30]. However, more meaningful outcomes should be studied before suggesting the use of this therapy in palliative care patients, such as subjective impression of effectiveness, comfort during therapy, fatigue caused by the therapy and PCF.

Expiratory muscle training (Table 2) is largely used in respiratory physiotherapy [48] but only two studies [18, 21] were found in patients with very severe stages of the disease. Both studies verified expiratory muscle training on cough effectiveness in patients with multiple sclerosis with respiratory muscle impairment. The results of the studies regarding this training were conflicting. One study presented an increase in PEMax and no significant change in maximum inspiratory pressure (PIMax) [21], while the other study verified improvement in PIMax, subjective cough effectiveness assessment and FVC and no change in PEMax [18]. Nevertheless, expiratory muscle training might be a suitable therapy only in very specific situations in palliative care, since an adequate nutritional balance and a long-term adherence to the therapy are essential to a clinically significant strength improvement [49], which are not usually possible in this population.

## Discussion

This paper systematically reviewed literature on interventions used to manage the presence of respiratory secretion. Nevertheless, there was limited evidence for most of the interventions and most were restricted to neurological and neuromuscular patients.

The low number of randomized controlled trials is similar to other reviews of symptom control in palliative care. Moreover, some interventions (such as tracheotomy) may be considered essential or usual care to patients despite the absence of evidence and therefore not providing this therapy to a group of patients in randomized controlled trial would be considered unethical.

The included randomized controlled trials and cross-over trials had a low overall methodological quality. Although some methodological quality criteria are usually not achievable by non-pharmacological interventions, such as blinded therapists and patients, some criteria are possible to be met, which affects the trust in the results.

Although diseases in advanced stages, such as dementia and cancer, do often result in the occurrence of discomfort due to respiratory secretion, no studies regarding these diseases were found, probably due to the reduced number of studies focusing patients’ comfort and symptom control in comparison to studies with therapies to control the advance of these diseases. There were only a limited number of studies on diseases where this condition is usually found [4], such as respiratory diseases. Neuromuscular diseases [45], such as Duchenne muscular dystrophy and amyotrophic lateral sclerosis, were more frequently studied.

Most therapies found in this review could be applied in palliative care situations, however more studies must be conducted to strengthen the evidence and broaden the populations that could be treated, especially in cancer. In addition, no studies were found which assessed drug therapy to relieve this symptom, which suggests that the use of anti-muscarinic drugs and mucolytics are based in studies that included patients with lower severity of the disease, or patients with death rattle.

More research is required for a better understanding of the efficacy of pharmacological and non-pharmacological therapies used in respiratory secretion management in patients in palliative care. The studies should be conducted with patients who may die in the following year, but are not so close to dying that respiratory secretion is considered to be treated only due to the sound it produces.

Additionally, none of the studies presented as the main outcome a patient-reported outcome measure (PROM), such as comfort and subjective effectiveness impression. These PROMs are critically relevant in authors’ opinion, since they capture the preferences and impressions of the patient, which is essential when assessing a palliative intervention [50]. However, these PROMs were assessed as secondary outcomes to MAC and MIE interventions, and for both PROMs, they were considered acceptable by patients. Interventions to improve mucus clearance and to increase cough effectiveness were not assessed regarding subjective impression of effectiveness or comfort.

Therapies that most probably suited palliative care patients are MAC and MIE as they both promote expectoration, chest physiotherapy and percussive ventilation applied by non-invasive methods to improve mucus clearance. Therapies that simply improve voluntary cough effectiveness are less likely to be used in this context. Drug therapy, such as mucolytics and anti-muscarinic agents should also be studied to verify their short and long term effects. In addition studies that assess the subjective experience of treatment by patients are required.

### Study limitations

Understandably this review has some limitations. Initially, the review was not prospectively registered. Moreover, a meta analysis was not done due to the large number of outcomes and therapies and the difference in methods among the studies would have affected this meta analysis; however, a broad overview of therapies which could be used in palliative care were identified, which can guide future steps in the research field. Moreover, the identification of studies that included patients who could be in a palliative care treatment were done using severity indices [51], such as forced expiratory volume in the first second in respiratory diseases and respiratory muscle impairment in neuromuscular and neurological diseases. Nevertheless this would be difficult to do, as few of the studies defined the population as palliative. Instead we included studies with patients suffering with an advanced disease akin to a palliative care population.

## Conclusion

Therapies, such as MAC, MIE and percussive ventilation, which aim to deal with respiratory secretion, can be used in palliative care of specific diseases. Nevertheless, the evidence still needs to improve in order to identify which alternative is the best.

## Abbreviations

ALS, amyotrophic lateral sclerosis; CF, cystic fibrosis; COPD, chronic obstructive pulmonary disease; EMT, expiratory muscle training; FEF_25–75%_, mean forced expiratory flow at 25–75 % expiratory period; FES, functional electrical estimulation; FEV_1_, forced expiratory volume in the first second; FVC, forced vital capacity; MAC, manually-assisted cough; MIE, mechanical insufflation-exsufflation; MS, multiple sclerosis; NMD, neuromuscular disease; OCEBM, Oxford centre for evidence-based medicine; PCF, peak cough flow; PEMax, maximum expiratory pressure; PEP, positive expiratory pressure; PIMax, maximum inspiratory pressure; PROM, patient-reported outcome measure; SCI, spinal cord injury; SpO_2_, peripheral oxygen saturation; WB, work of breath

## Additional file

Additional file 1: Table S1. Search Strategy, **Table S2.** PEDro Score in Randomized Controlled Trials, **Table S3.** PEDro Score in Cross-over Trials. (DOCX 18 kb)
