# Supplementary material for: Benefits of interventions for respiratory secretion management in adult palliative care patients—a systematic review
Source: BMC Palliat Care. 2016 Aug 9;15:74. doi: 10.1186/s12904-016-0147-y (PMC4979117; doi:10.1186/s12904-016-0147-y)
Supplement: Additional file 1: Table S1. — Search Strategy, Table S2. PEDro Score in Randomized Controlled Trials, Table S3. PEDro Score in Cross-over Trials. (DOCX 18 kb) [file 12904_2016_147_MOESM1_ESM.docx]

**Table A: Search Strategy**

| **Set 1: Symptoms** | **Set 2: The population** | **Set 3: Interventions** |
| --- | --- | --- |
| **MESH headings** | **MESH headings** | **MESH headings** |
| Bronchi/secretion | Terminal care | Expectorants |
| Trachea/secretion | Hospice care | Cholinergic Antagonists |
| Respiratory Mucosa/secretion | Palliative care | Drug Therapy |
| Respiratory insufficiency | **Free-text keywords** | Physical Therapy Modalities |
| Cough | Advanced | Patient Positioning |
| Sputum | "Life limiting" | Respiratory Therapy |
| **Free-text keywords** | Bed | Suction |
| Hypersecret* | hospice | **Free-text keywords** |
| sputum | palliative | “Airway clearance technique” |
| mucus | supportive | “Positive-Pressure Respiration” |
| “respiratory secret*” | "end stage" | “Mechanical insufflation-exsufflation” |
| “bronchial secret*” | severe | “Cough Assist” |
| “airway secret*” | terminal | Physiotherapy |
| “tracheal secret*” | "end of life" | “In-exsufflation” |
| “death rattle” | Death |  |
| “respiratory sound*” | Dying |  |
| phlegm |  |  |
| bronchorrhea |  |  |
| cough* |  |  |
| *MESH terms exploded* | *MESH terms for Hospice care and Palliative care exploded but not terminal care* | *MESH terms exploded* |
| *All terms combined with OR* | All terms combined with OR | *All terms combined with OR* |
| Sets 1 AND 2 AND 3 combined and then limited to Human and Adult | | |

**Table B: PEDro Score in Randomized Controlled Trials**

| **Study** | **Criteria** | | | | | | | | | | | **Total** |
| --- | --- | --- | --- | --- | --- | --- | --- | --- | --- | --- | --- | --- |
|  | **1** | **2** | **3** | **4** | **5** | **6** | **7** | **8** | **9** | **10** | **11** |  |
| **Pillastrini et al (2006)^17^** | X | X | 0 | 0 | 0 | 0 | 0 | 0 | 0 | 0 | 0 | 1 |
| **Gosselink et al (2000)^18^** | 0 | X | 0 | X | 0 | 0 | 0 | X | 0 | X | X | 5 |
| **Chaisson et al (2006)^19^** | X | X | X | X | 0 | 0 | 0 | X | 0 | X | X | 6 |
| **Christensen et al (1990)^20^** | X | X | 0 | X | 0 | 0 | 0 | 0 | 0 | X | X | 4 |
| **Smeltzer et al (1996)^21^** | 0 | X | 0 | X | 0 | 0 | 0 | 0 | 0 | X | X | 4 |
| **Rafiq et all (2015)^22^** | X | X | 0 | 0 | 0 | 0 | 0 | X | X | X | X | 5 |
| **Percentage** | 66,7% | 100,0% | 16,7% | 66,7% | 0,0% | 0,0% | 0,0% | 50,0% | 16,7% | 80,0% | 80,0% |  |

**Table C: PEDro Score in Cross-over Trials**

| **Study** | **Criteria** | | | | | | | | | | | **Total** |
| --- | --- | --- | --- | --- | --- | --- | --- | --- | --- | --- | --- | --- |
|  | **1** | **2** | **3** | **4** | **5** | **6** | **7** | **8** | **9** | **10** | **11** |  |
| **Senent et al (2011)^21^** | X | X | 0 | 0 | 0 | 0 | 0 | 0 | 0 | X | X | 3 |
| **Winck et al (2004)^22^** | X | 0 | 0 | X | 0 | 0 | 0 | X | X | 0 | X | 4 |
| **Bach**  **(1993)^23^** | 0 | 0 | 0 | X | 0 | 0 | 0 | X | 1 | 0 | X | 4 |
| **Sancho et al (2003)^24^** | X | 0 | 0 | X | 0 | 0 | 0 | X | X | X | X | 5 |
| **Chatwin, et al (2009)^25^** | X | X | 0 | 0 | 0 | 0 | X | 0 | 0 | X | X | 4 |
| **Chatwin et al (2003)^26^** | 0 | X | 0 | 0 | 0 | 0 | 0 | 0 | 0 | X | X | 3 |
| **Lacombe, et al (2014)^27^** | X | X | 0 | X | 0 | 0 | 0 | X | X | 0 | X | 5 |
| **Linder**  **(1993)^28^** | X | 0 | 0 | X | 0 | 0 | 0 | X | X | X | X | 5 |
| **Toussaint et al (2003)^29^** | 0 | X | 0 | 0 | 0 | 0 | 0 | X | 0 | X | X | 4 |
| **O’Connell, et al (2011)^30^** | X | 0 | 0 | 0 | 0 | 0 | 0 | X | X | X | 0 | 3 |
| **Percentage** | 70% | 50% | 0% | 50% | 0% | 0% | 10% | 70% | 60% | 70% | 90% |  |

Scale available on: <http://www.pedro.org.au/english/downloads/pedro-scale/>
